# Why were "starvation diets" promoted for diabetes in the pre-insulin period?

**DOI:** 10.1186/1475-2891-10-23

**Published:** 2011-03-11

**Authors:** Allan Mazur

**Affiliations:** 1 Maxwell School of Citizenship and Public Affairs, 435 Crouse-Hinds Hall, Syracuse University, Syracuse, NY 13244, USA

## Abstract

In the decade before the discovery of insulin, the prominent American physicians Frederick Allen and Elliott Joslin advocated severe fasting and undernutrition to prolong the lives of diabetic patients. Detractors called this "starvation dieting," and some patients did indeed starve to death. Allen and Joslin promoted the therapy as a desperate application of animal experimentation to clinical treatment, and texts still describe it that way. This justification was exaggerated. The public record contains only the briefest account of relevant animal experiments, and clinical experience at the time provided little indication that severe undernutrition had better outcomes than low carbohydrate diets then in use.

## Review

During the first third of the 20^th ^century, Frederick M. Allen and Elliott P. Joslin were among the most prominent diabetes specialists in America. Joslin, the elder man, encouraged Allen's early experimental studies, and the two physicians rapidly converged on the belief that severely calorie-restricted diets were the best therapy for diabetes. For a brief period, from 1915 until the introduction of insulin in 1922, they promoted what have been pejoratively called "starvation diets" - diets based on repeated fasting and prolonged undernourishment -- as the most advanced treatment for diabetes mellitus, not as a cure, but for relief of symptoms and maximum extension of life.

There is no doubt that fasting usually reduces glucose levels in diabetics, but prolonged calorie-restricted diets introduced new hazards, most obviously death by starvation, which Allen and Joslin euphemistically called "inanition." It is accepted today that calorie restriction is beneficial for diabetics who are overweight. But for those of normal or lower weight, it was already understood in Allen's time that severe calorie restriction could lessen resistance to infection, and for children it might stunt growth. Fasting and undernourishment therapy was a balancing act, overseen by a specialist while the patient remained under close supervision in a hospital or clinic, and prone to backsliding by patients who returned home. The permanently calorie-restricted diet was unpleasant, difficult to maintain, and enervating, with the result that many patients withdrew from the regimen. Observers wondered if such privation was too high a price for a modest extension of poor quality life. Joslin commented retrospectively, "We literally starved the child and adult with the faint hope that something new in treatment would appear...It was no fun to starve a child to let him live" [1].

Allen's most famous patient was Elizabeth Hughes, the daughter of Charles Evans Hughes, Governor of New York, Republican candidate for the presidency in 1916, and finally Chief Justice of the U.S. Supreme Court. Elizabeth developed diabetes in 1919 at age 11, her height then 4" 11 1/2", her weight 75 pounds. She was treated initially by Dr. Allen who put her on a week of fasting followed by a diet of 500 calories daily with one fast day per week, bringing her weight down to 55 pounds. Freed of glycosuria (sugar in the urine), her diet was raised to 1,250 calories, except on fast days, and her weight rose above 60 pounds. At that time, most child diabetics died from coma within months to a few years of diagnosis [2]. It is difficult to judge how much Allen's regimen prolonged Elizabeth's life. She disliked the treatment as well as Dr. Allen, but she adhered to the diet with oversight from her nurse companion.

Though an ideal patient, Elizabeth deteriorated seriously by the winter of 1921/22 when she weighed 45 pounds. Her mother pleaded with Canadian doctor Frederick Banting, a recent discoverer of insulin, to include Elizabeth as a trial patient. This was a marvelous success. Elizabeth regained weight, eventually graduating from Barnard College. She married, had three children, and was active throughout her life in civic affairs, all the while on insulin, until she died of pneumonia in 1981 at the age of 73. Few of her friends knew she was diabetic [3].

Allen knew that carbohydrates are the component of the diet that most affects blood glucose. Many practitioners of the time, and earlier, prescribed an "animal diet" or other low carbohydrate variation to clear glycosuria. Critics of the Allen-Joslin therapy claimed as much success, without near-total food privation, using diets low on carbohydrates but allowing more fat calories [4,5]. Why then did Allen -- and Joslin following him -- so severely restrict the caloric intake of their patients, nearly starving them?

## Allen's Early Work

Frederick Madison Allen (1879-1964) graduated from the University of California at Berkeley and completed his M.D. there in 1907. Moving to Baltimore, he contacted one of his fraternity brothers, now at Johns Hopkins, and spent a few weeks performing surgery on dogs, meeting professors, and looking for a good research problem but without success. Moving to Boston, he spent the years 1909 to 1912 as a teaching fellow at Harvard Medical School, conducting experiments on cats and dogs, largely at his own expense. An austere man, he describes himself in an unpublished memoir in the private collection of Alfred Henderson, Bethesda MD, as living like a hermit, continually working seven days a week.

Allen produced diabetes in his animals by partial pancreatectomy, the degree of disease depending on how much pancreas was removed. According to Alfred Henderson, a friend and biographer of Allen, "There resulted from this experimental period a mass of manuscript material, all written in longhand which appeared capable of considerable improvement. No publisher could be found to wade through his crude manuscript" [6]. With a subsidy from Allen's father, Harvard University Press published the manuscript as *Studies Concerning Glycosuria and Diabetes*, which ran 1,179 pages. In this book, Allen gives no prescription for calorie deprivation as a therapy for human diabetes, nor does he describe any felicitous effect of starvation on his animal subjects. To the contrary, Allen occasionally reaches conclusions that seem counter to his later position, e.g., "Fat feeding is not to be feared in diabetics" [7].

## Promoting Starvation

In 1913, the year his book was published, Allen left Harvard and was appointed a nonresident assistant physician to work on diabetes in the newly established Hospital of the Rockefeller Institute in New York [8]. In his memoir written years later, Allen recalled,

I quickly followed up the first clue from my Harvard work, proving that diabetes in partially depancreatized dogs, which was too severe to be controlled on a customary protein-fat diet or on any diet while the animals were fat, could be controlled and kept controlled by starving them and then dieting so as to keep them thin...Within a few months I was able to ask for human patients.

The first public statement of his treatment that I can locate was read at a session of the American Medical Association in June 1914, where Allen claims new results: If enough of a dog's pancreas is removed, glycosuria occurs even on meat feeding. In some of these dogs, a few days of fasting will make their urine sugar-free. If feeding of protein and fat is then begun cautiously, only enough to maintain the animal in its thin condition, such dogs remain free from diabetes. His longest experiment to date was on a dog kept free from glycosuria for six months and still alive. Allen cautioned, "If an attempt is made to increase the weight of such an animal, glycosuria soon appears and must be checked by renewed fasting. Such dogs, though very thin, are vigorous and lively" [9].

Allen presents his results anecdotally, giving no quantitative data, which was not unusual in presentations of that era, but he does not even report the number of animals used. It would have been impossible to even intuitively assess the reliability of his results or the implied differences between experimental and control animals, or if indeed there were control animals. He regards dogs as adequate models for human diabetics, and he functionally equates the dog's surgically reduced pancreas with human diabetes [also see [10]].

Allen adds that he has treated a "limited" number of patients by prolonged fasting and calorie restriction. "The results obtained indicate that the same method employed in rendering the diabetic dog free of glycosuria and prolonging its life is efficacious in eliminating glycosuria and acidosis in the human patient" [9]. Five years later Allen published detailed case histories, which show that he had by then treated only three diabetic patients, none longer than four months [11].

By fall, Allen had treated eight patients, and the hospital director thought his results sufficient to justify undertaking this work on a larger scale [12]. Allen envisioned diabetes occurring when a predisposed pancreas is pushed over the edge by excessive diet. The "overstrained" pancreas could no longer process glucose adequately, causing sugar to appear in the urine. Conversely, Allen thought an overstrained pancreas is rested by diminished diet, and that this respite may gradually strengthen its functioning. "The attempt to put on weight, according to the time-honored traditions of diabetic treatment, is one of the surest ways of bringing back all the symptoms and sending the patient downhill" [13].

A year later, addressing another medical group in May 1915, Allen is more emphatic. Reporting no new experiments but referring generally to surgically-produced diabetes in animals, he notes that the measures ordinarily used in human diabetics, namely brief fasting and carbohydrate restriction, may be insufficient to keep dogs sugar free. In severe cases, "the initial fast must sometimes be measured in weeks rather than in days. The subsequent diet must be such as to keep the animal at a low level of weight...If glycosuria is prevented, the animals may remain lively and strong though thin...The treatment of [human] diabetes at the Rockefeller Hospital has been based upon these animal experiments" [14].

Allen had by this time treated 44 patients at Rockefeller, "chosen as the most severe cases...and representing a sufficient variety as respects age, social condition, and other factors." He claims to have cleared up glycosuria with an initial fast, sometimes lasting as long as ten days.

Broadly speaking, freedom from glycosuria seems attainable in all cases of uncomplicated human diabetes before there is danger of death from starvation...After the fasting patient has been completely sugar-free for one or two days, feeding is begun...and the tolerance of the patient for carbohydrate, protein, and fat is determined...[T]he diet is governed...by the amount of each food that can be given in each individual case while keeping the urine clear [14].

It is difficult to tell precisely what the fate of Allen's 44 patients was from the closing statement in his May 1915 talk: "Among the patients treated thus far, during a variable number of months in the hospital and at home, spontaneous downward progress has not yet been observed." My tabulation of these cases, which were later published [11], shows that of 41 patients whose condition was known at the end of 1917, 46% were dead or in one case "nearly dead."

Allen was deeply committed to his new regimen, often interpreting comas or deaths to lapses by patients from their prescribed diets. He claimed repeatedly that his therapy was based on his animal experimentation, without publically reporting these experiments in detail, nor am I aware of anyone replicating his results. An article in *The New York Times *of February 13, 1916, says Allen's new therapy was based on animal experiments performed at the Rockefeller Institute by I. Kleiner and S. Meltzer, but was not true [15].

Historian Michael Bliss characterizes Allen as a "stern, cold, tireless scientist, utterly convinced of the validity of his approach... His therapy for diabetes seemed immensely hard-hearted in the extreme cases, and met much resistance from diabetics, their families, other physicians, and other workers at the Rockefeller Institute" [16]. To emphasize his point, Bliss cites Case 4, one of Allen's first patients at Rockefeller, a blind twelve-year-old boy already in bad shape when first seen in 1914, seven years after onset. Allen experimented with various diets and periods of fasting and was puzzled by unaccountable glycosuria unrelated to the known food intake. Allen remarks in his write-up,

It seemed that a blind boy isolated in a hospital room and so weak that he could scarcely leave his bed would not be able to obtain food surreptitiously when only trustworthy persons were admitted. It turned out that his supposed helplessness was the very thing that gave him opportunities that other persons lacked.... Among the unusual things eaten were tooth-paste and bird-seed, the latter being obtained from the cage of a canary which he had asked for. Also his mother and his governess on visiting him sometimes brought lunch, which was kept in a closet, supposedly without his knowledge; nonetheless, in the short intervals when he was unwatched, he managed to find it and remove such articles as might not be missed. These facts were obtained by confession after long and plausible denials.

This boy died after four months of treatment [11].

In his memoir, Allen evaluates members of the Rockefeller staff as either for or against him. Dr. Edgar Stillman, who had direct care of patients while Allen devoted time to his animal experiments, was "incapable" and "useless," later part of the opposition that would take over control of the diabetes ward. Dr. Reginald Fitz (son of Reginald Heber Fitz, a long-time professor at Harvard Medical School who was one of Joslin's early collaborators [17]), worked with the hospital's diabetic patients in a "competent and agreeable" manner, but "Dr. Fitz, having a career before him, was too circumspect to be allied with me on the losing side and made his friendships on the stronger side.".

Bliss suggests that Allen's determination to apply his methods ruthlessly led to a decision by the Rockefeller Institute to take away his control of the diabetes clinic. Allen's friend, Alfred Henderson, gives a more sympathetic portrait but a congruent reason for departure:

Initially a loner in his work and in his beliefs, days and nights became too short for the experimental animal work and the management of often severely ill patients who needed close attention and frequent laboratory tests. In addition, he found it necessary personally to supervise the hospital kitchen and dietary service, which were not inclined to take on the added burden of [Allen's demands, including]...the thrice-boiling of vegetables to reduce carbohydrate, one of Allen's innovations to become universally practiced... [The] Director of the Hospital...seemed disinterested in Allen's pleas for additional time for repeated experimental confirmation of his ideas. Things went from bad to worse in his relationship to authority, and he soon found his activities restricted to the animal laboratory [6].

In 1917, as the U.S. entered World War I, the head of the Institute, Simon Flexner, informed Allen that he would be a captain in charge of a diabetic service at Lakewood, New Jersey, effectively terminating his employment. Given his checkered career to this point and the dubious scientific basis for his treatment, one may wonder why his therapy would soon become so popular and Allen so prominent.

## Joslin's Enlistment

Elliott Proctor Joslin (1869-1962) was born in small town Oxford, Massachusetts into a prosperous family of religious Congregationalists. He received his M.D. from Harvard Medical School. After further training, he opened a private practice in Boston in 1898, the first doctor in the U.S. to specialize in diabetes, and was the founder of today's Joslin Diabetes Centers. His book, *The Treatment of Diabetes Mellitus *[18], went through many editions, indicative of Joslin's prominence.

A man of deep faith in religion and science, Joslin was frugal but immaculate in dress, rising early, working long hours, eating sparely. According to Chris Feudtner, who studied Joslin's long career, he had a spry body, a serene sense of discipline, and a stern New England conscience. He combined a "gentlemanly manner with a showman's knack and a preacher's zeal, always looking to spread his message of diabetic care" [19].

In 1915 the well-established Joslin generously credited the younger Allen for great progress that the period 1914-15 had seen in the treatment of diabetes mellitus:

The advantage of maintaining the urine sugar-free has been universally recognized, but all have conceded that this was impossible without danger from acidosis and inanition. Fasting and a low diet have been known, but it is only fair to give Allen the credit of first to see the therapeutic significance of inanition upon a severe case of diabetes, second to prove upon diabetic dogs that prolonged fasting would render them sugar-free, and third to have the courage of his convictions and apply this principle to human diabetes. Thanks to Dr. Frederick M. Allen we no longer nurse diabetics - we treat them! [20].

I see no reason to doubt that Joslin was genuinely impressed by Allen's therapeutic achievements, but there was as well a preexisting relationship between the men going back to Allen's time at Harvard. Joslin was an assistant professor at Harvard Medical School while Allen was a teaching fellow, and they knew one another if only for their shared specialization in diabetes. Dr. Alfred Henderson, who knew both men in their later years, told me that they communicated about patients while both were at Harvard. Also, they were fraternity brothers, members in their respective undergraduate colleges of Alpha Delta Phi, the fraternity of John D. Rockefeller, Jr., and Theodore and Franklin Roosevelt [21]. In the preface to his Harvard University Press book, Allen acknowledges his indebtedness to Joslin for "good offices in connection with publication." As a fellow at Harvard, Allen received a $300 grant for his diabetes work from the medical school's Proctor Fund, established by bequest of Ellen Osborne Proctor for the study of chronic diseases. Ellen Proctor was Elliott Proctor Joslin's aunt, and he was instrumental in establishing the fund [17]. In the preface to the 1917 edition of his text, Joslin writes, "I can never repay Dr. Allen's many and continued kindnesses or sufficiently acknowledge the inspiration which his fruitful and persistent work awakens" [18].

As a consequence of having a large practice in diabetes but few effective tools, Joslin had by 1916 lost 62 patients under the age of fifteen, with coma always the cause of death [22]. In adopting Allen's treatment, first removing fat, then protein, then carbohydrate from the diet, Joslin forestalled hyperglycemic crises and ketoacidosis that are known today to induce diabetic coma [23]. Quickly noticing the improvement, especially among his child patients, Joslin reported "the gratifying fact" that in the past year, "48 cases [probably mostly adults] have been treated by me in hospitals without a death" [22]. Two years later he would comment, "Whereas formerly the prognosis for children less than ten years of age was measured in months, today it is rare for a child to live for less than one year.". Furthermore, Joslin saw a pragmatic benefit for general practitioners: "The greatest advantage of the fasting treatment introduced by Dr. Allen lies in its simplicity and in the removal of the need for quantitative urinary examinations" [18].

His clinical experience deeply impressed Joslin: "For myself, I consider the impression which I have thus obtained of far more value than any statistics which my records afford, but it is almost my duty to present these as well." He summarized his cases in tables but was not an adept statistician by the standards of the time. (Even his arithmetic is incorrect.) His Table One shows 85% of his new patients still alive but only 80% of his fasted patients still alive, a puzzling comparison to affirm the value of fasting. Nonetheless, Joslin ends on an exuberant note: "I am coming to feel that coma no longer represents the culmination of the disease, but that it is an avoidable accident" [22].

In all his subsequent publications, Joslin praises Allen as the primary innovator in diabetic therapy. The second edition of his textbook, *Treatment*, opens with Joslin writing, "The advance in the treatment of diabetes, which began with the introduction of fasting by Dr. F. M. Allen, continues, and statistics are now available to show it. So-called acutely fatal diabetes is disappearing and the first year of diabetes is no longer, as was only too recently the case, the diabetic's danger zone. Already I have quite a series of patients who have outlived their normal expectation of life at the age of onset of their diabetes." And later in the text:

Looking back upon the treatment of diabetes before Allen's introduction of prolonged fasting, it really seems...that our patients were nursed rather than treated. Only those who have cared for many patients by the older methods can appreciate the advance which Allen has given to diabetic therapy [18].

In February 1916, *The New York Times *reported a symposium held at the New York Academy of Medicine on the "Allen Plan." Whether or not Joslin had a hand in setting up this event, he gave it his imprimatur. Dissenters also participated, but the story's headline, "Radical New Method of Treating Diabetes," suggests that it was a platform for Allen, who told his colleagues, "Many of our patients run up the eight flights of our stairs at the hospital of the institute twenty times a day. Then they walk eight or ten miles in the open air. They also skip the rope and toss medicine balls. We are making athletes of them, thin as they are, and surely it can at least be said of them they are not neurasthemic" [24].

The next year, Joslin published data tables based on 1,187 cases of diabetes he saw personally between 1893 and 1916. He claimed to show with these data that since 1915, when "modern therapy" was first applied, outcomes have improved. In one such display, he compares 408 ultimately fatal cases he saw in the period 1893-1915 with 500 ultimately fatal cases he saw in the period 1893-1916 (i.e., the latter group contains the 408 fatalities of the former group plus 92 fatalities since 1915). Joslin emphasizes that 16.9% of the 1893-1915 fatalities died within one year whereas only 14.8% of the 1893-1916 fatalities died within one year. He concludes, "A change in mortality from 16.9 per cent in the first year of the disease to 14.8 percent does not seem large, but these figures, as do those for the subsequent years, understate the real improvement in treatment...These tables, to my mind, present irrefutable evidence of the improvement in the treatment of diabetes" [18].

This is muddy statistical reasoning, even by the standards of his time. First, the two groups Joslin compares (1893-1915 vs. 1893-1916) contain mostly the same cases. Second, in combining all his cases from 1893 to 1915, Joslin implicitly assumed that there was no gradual improvement in the mortality of his patients over these years, but elsewhere he notes that adults previously unsuspected of having diabetes were by then being identified in examinations for insurance policies, and his practice increasingly included older patients who generally lived longer from onset. To address both of these concerns, a better comparison would have been one-year mortality for patients newly seen in 1915-16 (after the introduction of Allen's therapy) with the one-year mortality for patients newly seen in 1914-15. Third, Joslin acknowledges that the change in mortality "does not seem large." If he had been writing in today's language, he might have said the 2% difference "is not statistically significant." It would be unreasonable to hold Joslin to standards not then in place, but one can calculate that at least a 10% difference would be required for the Allen treatment to show a "significant" improvement in mortality at the now-conventional level of p = .05.

Poignantly, Joslin later adds "a discouraging nature [of the new treatment], for a new cause of death appears in my records - namely inanition, and to this 3 cases succumbed. Inanition, like coma, may be rightly considered at present as evidence of unsuccessful treatment" [18]. Joslin blamed himself for these deaths.

## Allen Back in Civilian Life

By the war's end, Allen was a star in the diabetes community, championed by Joslin, with imitators and critics. In 1919 Allen's second book appeared as a Rockefeller Institute publication with his former associates at the hospital, Drs. Stillman and Fitz, as coauthors, though Allen says in his memoir that he wrote the entire text.

Again a civilian, Allen purchased an abandoned estate with a golf course in suburban Morristown, New Jersey, converting it into a hospital and laboratory. His Physiatric Institute opened in 1920, its Greek-derived name indicating treatment through natural means. Rates varied with the quality of accommodations, whether wards or luxurious rooms. Diet therapy was individualized, requiring almost as many dieticians as nurses. Patients who were able could recreate on the grounds or the nearby town. Allen held complete control of the Institute, its medical, administrative, and fund raising aspects and, to the extent he could, its patients.

The Institute grew rapidly, holding one or two month-long teaching courses per year, drawing physicians from across the nation and disseminating calorie-restriction therapy. Soon young Elizabeth Hughes was brought for treatment, and her famous father became a supporter. After 1922, both Allen and Joslin were among the early adopters of insulin, but whereas Joslin's patient-care model flourished, Allen's operation contracted, especially after the crash of 1929. He was evicted from Institute property for defaulting on the mortgage [6].

## The Rockefeller Series

Allen, Stillman and Fitz treated about 100 diabetic patients in Rockefeller Hospital between February 1914 and December 1917, some admitted multiple times for an average of 69 days per admission. The 1919 book describes 76 cases, most in considerable detail [11]. Like many clinicians of his day, Allen did not think statistically but rather in terms of individual case outcomes. Here, abbreviated, are contrasting examples of juvenile patients, the first having a poor outcome, the second good:

Case 51: A seven-year-old boy was first seen in 1915, a year after onset, his height at admission 121 cm, his weight 18.3 kg. In August 1916 the boy, weighing 17 kg., was discharged from his second stay at the Institute on a prescribed 700 calorie diet with a weekly fast day. He died two months later; details were not obtained. "The essential cause of trouble lay in the home conditions of an uneducated Polish laboring family... [N]o obvious recuperation or repair of the assimilative function was displayed by this child under these circumstances."

Case 76: A four-year-old boy was first seen in 1917, three weeks after onset, his height at admission 107 cm, his weight 15 kg. He was discharged weighing 14.4 kg on a prescribed 980 calorie diet with a weekly fast day. This boy was still well in 1922 when he was switched from diet therapy to insulin. "[T]reatment has been followed with the utmost fidelity... This boy has remained constantly faithful to diet while attending school and carrying on all activities normal for his age."

There is a tautological character to Allen's *post hoc *interpretations. He explains a case with poor outcome as due to dietary lapse, often the patient's or his family's own fault. He explains a good outcome as the result of faithfully sticking to diet. How credulous of Allen to believe that Case 76, a normally active little boy among his school friends, remained constant to his Spartan diet! A page later he complains of "patients supposedly following a diet at home [who]... seem most trustworthy [but] sometimes become proficient in violations of diet which are hard to detect."

Looking over his Rockefeller cases, Allen concludes, the acutely threatening symptoms of diabetes have been controlled by the present treatment in a successful and radical manner which bears comparison with the most powerful therapeutic measures for any acute or chronic disease; but the diabetes is not cured, and downward progress occurs in practically all potentially severe cases unless the same principle of limitation of the total metabolism and body weight is adequately observed at all times...Spontaneous or inevitable downward progress has generally been either absent or not demonstrable in typical cases of diabetes of even the worst type...[It is true that] unduly numerous examples of downward progress have occurred in the present series. These were attributed to blunders and mismanagement in the application of the principle of treatment [11].

The series of cases presented for study in the 1919 book begins with his first diabetic patient, seen in February 1914, and runs through those seen by June 1916, "so as to insure at least 16 months of observation in every published case." For various reasons that I have accepted at face value, he ignores this scope condition by adding eight cases first seen after June 1916, giving a total of 76 cases. In the main text, Allen reports their condition as known in late 1917. Footnotes added prior to publication update four cases to February 1918.

Allen never made claims of a miracle cure and notes forthrightly that 43% of his 76 cases were dead by late 1917. He acknowledges four deaths from starvation (inanition) under treatment.

The result of prolonged fasting that so impressed Elliott Joslin was the reduction of coma (and consequent death), especially in children. Allen reports the occurrence of "full" or "incipient" coma in 21 of his cases. He usually treated these by fasting, with fourteen recovering. While a fully comatose patient is unmistakable, Allen acknowledges that it is difficult to judge when a patient is in "incipient coma" if the condition never deteriorates to full coma. Those incidents that he labeled "incipient" account for most of his recoveries. Furthermore he has no non-fasted control group, so he cannot evaluate differential rates of coma with and without fasting. Lacking quantitative data, we might trust Joslin's experienced clinical eye in seeing a reduction in coma with fasting.

Did severe calorie restriction prolong life more than the small extension gained by sidestepping a particular coma-and-death event? This question is difficult to address without a proper control group, but Allen's own data seem to belie the optimism with which he promoted the undernourishment regime. The 44 patients of whom he spoke in 1915 are the first 44 in the Rockefeller series. He knew the condition of 41 of these people in October 1917, by which time 46% were dead. Eleven among the 44 were under age 20 at first admission; 78% were dead by October 1917. (I made these calculations by combining data in Table One of Chapter II and Table One of Chapter VII in [11].)

Indeed, when Allen spoke about the 44 in May 1915, he knew that 20% were already dead. Nine of those still alive had been under his care less than five months. One wonders about his candor in opaquely concluding, "Among the patients treated thus far..., spontaneous downward progress has not yet been observed."

Turning to the full series of 76 cases, Allen tabulates mortality by age of admission to Rockefeller. My Table 1, which simplifies and updates Allen's tabulation to February 1918, shows a relationship already well known between age and mortality. Although clinicians of the time did not distinguish types 1 and 2 diabetes, they knew that the disease almost inevitably meant death for young children, if not in months then in a few years. Older people, whom we now know had predominately type 2 diabetes, could often manage fairly well. As Joslin commented, "In our enthusiasm for new methods it should not be forgotten that even in the past good results were obtained with the majority of [older] diabetics, and that gradual restriction of carbohydrate was the means employed" [18].

**Table 1:** Rockefeller Series: Mortality Rate by Age of Admission

Age at first admission to Rockefeller Hospital (years)	Number of patients	Percent known dead as of February 1918
0-9	8	75%

10-19	14	71%

20-29	14	64%

30-39	16	38%

40-49	13	31%

50+	11	18%

Lacking a proper control group, we cannot say whether or not Allen's undernourished patients fared much better than those on a more ample diet with restricted carbohydrates. Allen would emphasize that a quarter of those in the first decade of life remained alive. However, his two living patients below age ten (Case 73, age 3, admitted December 18, 1916; and Case 76, age 4, admitted March 9, 1917) were *ad hoc *additions to the series after the June 1916 cutoff date. The 3-year-old girl, under his care ten months, was already "weak and emaciated." The 3-year-old boy, his condition "good," was only eight months from onset of the disease. There is not much latitude here for optimism about the treatment.

Allen did not first see Elizabeth Hughes until 1919 when he was well situated at the Physiatric Institute, so she is not included among the 10 to 19-year-olds in the Rockefeller series. Four of 14 patients in her age category were still alive in February 1918. We are told in a footnote that one, an 11-year-old girl (Case 28), initially described in "good" condition, had a relapse and was referred elsewhere for treatment (p. 292). For a 19-year-old female (Case 62), Allen predicted "death from coma or inanition unless the patient is radically taken in hand and undernourished far more rigorously." The third, a 12-year-old boy (Case 64), is "emaciated" but alive. The fourth, a 15-year-old girl (Case 66), one year and seven months into treatment (two years from onset), "remains well and is pronounced by her mother the strongest and most energetic member of the family." We do not know if she survived until insulin became available.

## Conclusions

At the beginning of the 20^th ^century, physicians had no useful weapon against diabetes except the ability to easily measure sugar in urine. These tests became widely used in insurance examinations, uncovering a far higher incidence of diabetes in the adult population than previously recognized. Once diagnosed, the usual medical advice was for obese patients to lose weight and for all diabetics to reduce dietary starch and sugar. The difference between types 1 and 2 diabetes was not understood until the 1930s, but doctors had long known that in children the disease was usually fatal through coma, while it might be tolerated with little acute difficulty in some older people.

For a brief period, from 1915 until the discovery of insulin in 1922, prolonged fasting and permanent calorie restriction were championed in North America by Frederick Allen and Elliott Joslin. Offering hope for child diabetics and their parents, and for severely afflicted adults, the Allen Plan was quickly and widely adopted. This episode is worth examining for the view it gives us of the frail basis upon which new and extreme treatments could become common practice at that time.

Allen developed his therapeutic ideas in 1913 at Rockefeller Hospital, saying they were based on his experiments with partially pancreatectomized dogs. He doubtlessly did abundant work on animals at Harvard prior to 1913, but these results, published in detail, have no bearing on calorie restriction therapy. Later animal experiments at Rockefeller do relate to the treatment but are published (in an era before peer review) only sketchily and impossible to critically evaluate. Not even the number of comparison animals is reported, which may have been as few as two. If Allen did establish in dogs a reliable model for calorie-restriction therapy, he did not at the time support it with detailed results in the public record.

From 1914 to 1917 Allen, Stillman and Fitz treated about 100 patients in the diabetic ward of Rockefeller Hospital. Allen never said undernourishment was a cure for diabetes but did claim success in eliminating or avoiding glycosuria and acidosis, mitigating the worst outcomes of diabetes, and reinvigorating his patients -- if they adhered strictly to their diets. In the 1919 book, Allen describes 76 of these cases in detail, and while recording many deaths, nonetheless sees this record as verifying the efficacy of undernutrition. Writing before statistical analysis was established in the medical literature, Allen interpreted his clinical material on a case-by-case basis. He reliably saw calorie restriction as the reason for successful cases, and lapses from diet as the reason for failures.

Fasting and under-nourishment did free the urine of sugar and apparently reduced the incidence of coma-and-death in children. But child diabetics soon died from infections and other causes, now including inanition. It is uncertain whether calorie restriction extended life beyond the weeks or months gained from avoiding a particular coma event. My reanalysis of the Rockefeller series, showing very high mortality among children, provides little reason to think that it did. This judgment is obviously limited by the absence of a proper comparison group.

In reading Allen's publications through 1919, I got the impression that nearly all of the diabetic cases at Rockefeller Hospital from 1914 to 1917 were treated by his method. I was surprised to read a contrary recollection by Allen, abstracted from the discussion at a medical association meeting in 1921:

Contrary to my belief and wish, most of the diabetic patients in the Rockefeller Institute Hospital were given high calorie diets, and all the severe cases thus treated ended fatally. The only two juvenile patients who are alive today are the two who escaped this treatment and the sole known difference between them and the others is that their diets were kept relatively low in fats and calories. One of these two has lost tolerance seriously through surreptitious taking of fat, especially butter, and this decline of assimilation seems to have been halted by eliminating this error... Other clinical experience convinces me firmly that the attempt to give a high caloric diet or to build up the body weight too high with fat or any other food is injurious and leads to a fatal result in every genuinely severe case of diabetes [5].

It is impossible for me to say if this statement is factually correct, but Allen was adamant in restating this comparison the following year:

Unfortunately the great majority of these [Rockefeller] cases were subjected to reckless overfeeding with fat and total calories. The fact that only some of these milder cases have survived, and all the children and young persons and all the adults with severe diabetes have died under this treatment, stands as a sufficient warning against this method and serves sufficiently as a control of the results obtained under the method of caloric limitation which I have always advocated [25].

If we accept Allen's claim that undernourishment worked, it is worth asking if it worked any better than more filling low-carbohydrate diets. As critics Newburgh and Marsh put the issue,

A diabetic diet, in order to be satisfactory, must be capable of enabling the patient to lead a moderately active life for an indefinite period...[T]he severe diabetic may be kept sugar free by a sufficient reduction of his total caloric intake, but it is frequently necessary to reduce the total calories so much...that such patients suffer from slow starvation, and are quite incapable of earning a livelihood -- indeed many of them may be said to merely exist [4].

Newburgh and Marsh report on 73 cases, apparently mostly adults, admitted to the University of Michigan Hospital, where the state's physicians referred their severest cases as a "court of last appeal." On entering the clinic, patients were not fasted but placed on a diet of 900-1,000 calories, high in fat, low in carbohydrate. After being sugar free for one or two weeks, the diet was increased from 1,400 to 2,500 calories, depending on the needs of the person, most calories coming from fat, least from carbohydrate. Excepting three patients who died within a day of admission, and another who "went into coma after eating a bag of oranges brought by a relative," the urine of all patients became sugar free, none developed severe acidosis, and the investigators were "greatly impressed by the excellent condition of our patients months after leaving the clinic" [4].

I am not submitting the Michigan study as definitive, but it fortified the position of anti-starvation physicians who argued that carbohydrates were the main source of glucose in urine and blood, so it was unnecessary to starve patients as long as carbohydrate was limited, and for seriously ill children there was little that could be done in any case. Critics also claimed, as had Allen himself in 1913, that "fat feeding is not to be feared in diabetics" [7].

We cannot say whether Elizabeth Hughes would have lived until the advent of insulin if Allen hadn't nearly starved her to death. But it does appear, more generally, that undernourishment therapy was promoted as a treatment for diabetes without clearly supporting evidence from either animal or human studies.

Why, then, did the treatment and its promoter achieve such prominence? Joslin's support was critical for Allen to carry the day. This may have hung partly on the prior relationship between the men. Joslin did have some observational grounds for his enthusiasm. Prolonged fasting seemed to alleviate coma as the proximate cause of death in his pathetic child patients, even if it barely extended their lives.

Possibly the self discipline required by the Allen Plan appealed to the puritanical outlook of the two physicians. Allen and Joslin were very hard working, self disciplined men, physically trim and averse to excess or sloth. Both believed that diabetic patients must take charge of their own wellbeing. Allen often saw deteriorations in health as his patients' own fault for lapsing from their diets. Joslin proselytized that it was the diabetics' own responsibility to rigorously control their lives. Indeed, their ideology about proper lifestyle may have been as important for their advocacy as evidence-based effects.

The entire episode was brief. The primacy of diet diminishing once insulin became available, leading to the primacy of medication. Weight loss apart, there is still today no evidence-based consensus on proper diabetic diets [26,27]).

## Competing interests

The author declares that they have no competing interests.

## Authors' contributions

AM carried out all research for this article.
